# Inter-Network Interactions: Impact of Connections between Oscillatory Neuronal Networks on Oscillation Frequency and Pattern

**DOI:** 10.1371/journal.pone.0100899

**Published:** 2014-07-09

**Authors:** Oscar J. Avella Gonzalez, Karlijn I. van Aerde, Huibert D. Mansvelder, Jaap van Pelt, Arjen van Ooyen

**Affiliations:** Department of Integrative Neurophysiology, Center for Neurogenomics and Cognitive Research, VU University Amsterdam, Amsterdam, The Netherlands; Plymouth University, United Kingdom

## Abstract

Oscillations in electrical activity are a characteristic feature of many brain networks and display a wide variety of temporal patterns. A network may express a single oscillation frequency, alternate between two or more distinct frequencies, or continually express multiple frequencies. In addition, oscillation amplitude may fluctuate over time. The origin of this complex repertoire of activity remains unclear. Different cortical layers often produce distinct oscillation frequencies. To investigate whether interactions between different networks could contribute to the variety of oscillation patterns, we created two model networks, one generating on its own a relatively slow frequency (20 Hz; slow network) and one generating a fast frequency (32 Hz; fast network). Taking either the slow or the fast network as source network projecting connections to the other, or target, network, we systematically investigated how type and strength of inter-network connections affected target network activity. For high inter-network connection strengths, we found that the slow network was more effective at completely imposing its rhythm on the fast network than the other way around. The strongest entrainment occurred when excitatory cells of the slow network projected to excitatory or inhibitory cells of the fast network. The fast network most strongly imposed its rhythm on the slow network when its excitatory cells projected to excitatory cells of the slow network. Interestingly, for lower inter-network connection strengths, multiple frequencies coexisted in the target network. Just as observed in rat prefrontal cortex, the target network could express multiple frequencies at the same time, alternate between two distinct oscillation frequencies, or express a single frequency with alternating episodes of high and low amplitude. Together, our results suggest that input from other oscillating networks may markedly alter a network's frequency spectrum and may partly be responsible for the rich repertoire of temporal oscillation patterns observed in the brain.

## Introduction

Oscillations in electrical activity are observed in many brain networks, including the hippocampus [1], [2], prefrontal cortex [3] and visual cortex [4], [5], and occur in various frequency bands, ranging from fast gamma (40–80 Hz) to ultra-slow delta (0.1–1 Hz) [6], [7]. Network oscillations as revealed in extracellular field recordings are produced by the periodic and synchronized firing of large number of cells [8], [9], [10] and arise as a result of interacting populations of excitatory and inhibitory cells [8], [11], [12]. Oscillations have been linked to cognitive functions, such as attention [13], [14], [15], temporal binding [4], [16], [17], learning [6], [18], working memory [19], [20], [21] and memory consolidation [22].

**Figure 1:**
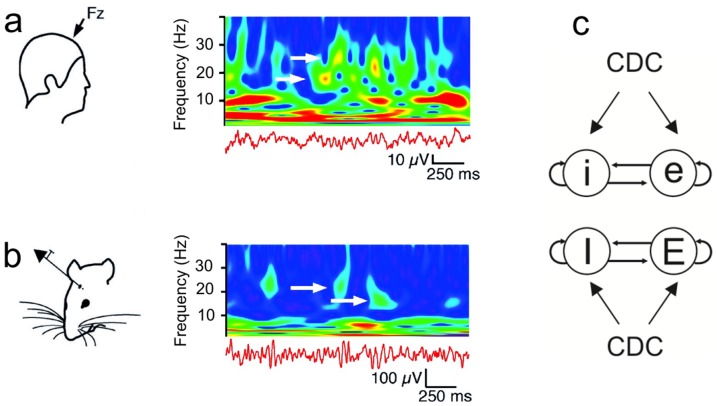
Distinct oscillation frequencies appearing intermittently in time, and schematic representation of model networks. **a.** Wavelet of EEG recordings in the frontal zone of the human brain, showing two interspersed, non-harmonic frequencies (17.7±0.8 Hz and 22.9±0.8 Hz; white arrows) in the beta range. Color indicates power of oscillations. When one of the frequencies has high power, the other oscillation frequency is absent or has low power. Adapted from [26] (Fig. 12 therein). **b.** Intracranial field recordings in the medial prefrontal cortex of awake rats show similar dynamics. The two main frequencies are 15.8±0.3 Hz and 22±1.7 Hz (white arrows). Adapted from [26] (Fig. 12 therein). **c.** Schematic representation of the two model networks, each consisting of a population of excitatory cells (e, E) and a population of inhibitory cells (i, I). The network generating slow oscillations is labelled with lower case letters, and the network producing fast oscillations is labelled with upper case letters. In each network, the inhibitory cells projected among each other and to the excitatory cells. Likewise, the excitatory cells projected among each other and to the inhibitory cells. In addition, both cell types received external input in the form of a constant depolarizing current (CDC). Furthermore, the cells of one network projected to the cells of the other network (not shown). The different inter-network connectivity schemes studied are shown in Fig. 2.

Ongoing oscillations display a rich repertoire of dynamical patterns [23]. The same neuronal network can express multiple oscillation frequencies at the same time [23], [24], [25], or the frequency can fluctuate over time, with distinct frequencies appearing intermittently (Figs. 1a, b) [26]. In addition, the oscillation amplitude can fluctuate, with episodes of high amplitude alternating irregularly with episodes of low amplitude [3], [26], [27], [28]. The origin of these complex oscillation patterns is unclear, but interactions between cortical layers may contribute.

Different cortical layers often produce distinct oscillation frequencies. For example, in association cortex, gamma and beta rhythms are generated in different cortical layers [23]. In the prefrontal cortex (PFC), layer 3/5 consistently oscillates at a higher frequency than layer 6 [26]. A similar distribution of oscillation frequencies occurs in visual cortical areas V1 and V2 [26]. Cortical layers are typically strongly interconnected with each other [29], [30], [31], but it remains poorly known how inter-network connections between oscillatory networks affect oscillation frequency and pattern [32].

Here, we investigated the impact of inter-network connections on oscillation frequency and pattern, examining whether these connections could account for complex temporal dynamics, such as coexistent frequencies and amplitude fluctuations, as observed in the PFC [26]. Most model studies on interacting oscillatory networks do not systematically explore inter-network connectivity, do not consider interactions between networks with different oscillation frequencies (but see [33]), or use mean field approaches to describe neural activity (e.g., [34], [35], [36], [37], [38]).

To investigate how one network can influence the rhythmic activity in another network, we built two model networks, each consisting of excitatory and inhibitory cells. One network was tuned to produce relatively slow oscillations on its own, and the other one was tuned to generate fast oscillations. We then systematically explored how adding uni-directional connections from the slow to the fast network or from the fast to the slow network affected the oscillatory activity in the fast or the slow network, respectively. We show that this afferent input can dramatically change the frequency spectrum in the receiving network and thereby generate a wide range of complex temporal patterns of oscillations similar to those observed in the PFC.

## Methods

To investigate how one network can influence the rhythmic activity in another network, we built two model networks in NEURON [39], each composed of a population of 80 excitatory (E) cells and a population of 20 inhibitory (I) cells (Fig. 1c). In general, a network of interconnected E and I cells can generate rhythmic changes in electrical activity [40]. The E cells activate the I cells, which in turn suppress the E cells, leading to periodic states of more or less synchronized spiking activity, both in the E population and in the I population. The amplitude or power of this oscillatory activity is determined by the number of synchronously firing cells. The oscillation frequency is determined by the average time between successive states of synchrony.

By selecting appropriate values for the IPSC decay constant of the inhibitory GABAA channel [2], [26], [40] (see section Networks), we tuned one network to produce relatively slow oscillations (about 20.4 Hz; this network is referred to as the slow network) and the other one fast oscillations (about 32.4 Hz; this network is referred to as the fast network). Note that these frequencies are non-harmonics of each other. They fall well into the range of frequencies reported for the hippocampus [41], [42], [43] and PFC [3], [26]. The difference in oscillation frequency is also similar to that observed between different subnetworks of the PFC [3], [26].

In addition to the connections within the slow and the fast network, one network also projected connections to the other network. The network that received input connections from the other network is referred to as the target network; the other network is called the source network. To keep the number of different inter-network connectivity schemes manageable, we did not include reciprocal connections between the two networks. By analysing the firing dynamics in the target network for a wide range of connectivity schemes, we investigated whether and how the source network perturbed the oscillatory activity in the target network and how this perturbation depended on the strength and pattern of connections from the target to the source network. The source code of the model will be made publicly available in the ModelDB database (http://senselab.med.yale.edu/modeldb).

### Cells

Both E and I cells were defined as one-compartment, conductance-based models, with a length and diameter of 20 µm. They contained the Hodgkin-Huxley Na^+^ and K^+^ channels, responsible for action potential generation, as well as leakage channels. The change in membrane potential *V* (in mV) was given by 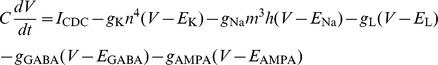



where *t* is time in ms; 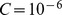 F/cm^2^ is the membrane capacitance; 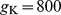 pS/μm^2^ and 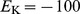 mV are the maximal conductance and reversal potential of the K^+^ channels; 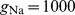 pS/μm^2^ and 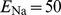 mV are the maximal conductance and reversal potential of the Na^+^ channels; and 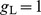 pS/μm^2^ and 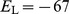 mV are the conductance and reversal potential of the leakage channels. Each cell received synaptic input from other cells, with 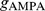 and 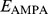 the synaptic conductance and reversal potential of the AMPA channels; and 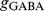 and 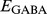 the synaptic conductance and reversal potential of the GABAA channels (for parameter values, see section Networks). In addition, each cell was stimulated by an external input in the form of a constant depolarizing current 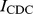
(see section External drive).

The dynamics of the gating variables *n*, *m* and *h* (collectively denoted by *z*) of the Na^+^ and K^+^ channels were given by
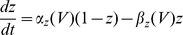



with 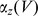 and 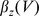 the voltage-dependent opening and closing rate constants. For the *n*, *m* and *h* variables, these functions were
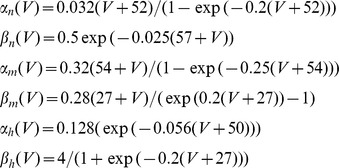



All parameter values were as in [44], and were the same for E and I cells. The only difference between E and I cells was that E cells projected to their target cells with excitatory glutamatergic AMPA synapses, and I cells with inhibitory GABAA synapses.

### Networks

The connectivity structure within each network was created by assigning to every cell a certain probability to connect to any other cell in the network. In addition, cells from the source network connected with a certain probability to any other cell in the target network.

Within each network, excitatory (E) cells were connected to inhibitory (I) and other E cells with probabilities 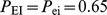 and 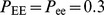, respectively. I cells were connected to E and I cells with probabilities 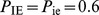 and 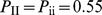, respectively. The first and the second letter in a subscript refer to the presynaptic cell and the postsynaptic cell of the connection, respectively. The upper and lower case letters indicate fast and slow network, respectively. A connection consisted of a single synapse with a synaptic conductance as described below.

The connectivity structure within each network was chosen on the basis of the following considerations. First, the use of connection probabilities prevents unrealistic all-to-all connectivity [45]. Second, the connection probabilities should be high enough to create a globally connected network rather than a number of isolated subnetworks. Third, the oscillations should be generated by a so-called PING (Pyramidal Interneuron Network Gamma) mechanism [40]. In this mechanism, which underlies the generation of most beta and gamma oscillations in the brain, the E cells (pyramidal cells) activate the I cells (interneurons), which in turn suppress the E cells, for a length of time that depends on the strength and the decay time of the inhibitory (i.e., GABAA) synaptic conductance. Once inhibition onto the E cells has subsided, the E cells can become active again, and the cycle repeats itself. Thus, oscillations are generated not by intrinsically oscillating cells (“pacemakers”) but by synaptic interactions within a network. The PING mechanism depends on strong connectivity from E to I cells, strong connectivity from I to E cells, and, to promote synchronous firing, connectivity from I to I cells [40].

The probabilities for the formation of connections between the two networks were based on the probabilities within each network, as follows: for the connections from inhibitory to excitatory cells, 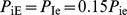; for the connections from excitatory to excitatory cells, 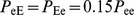; for the connections from inhibitory to inhibitory cells, 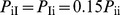; and for the connections from excitatory to inhibitory cells, 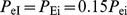.

For both excitatory and inhibitory synapses, the time course of the synaptic conductance was given by a mono-exponential function. For the excitatory AMPA synapses, the maximal conductances were 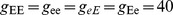 pS/μm^2^ and 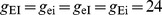 pS/μm^2^, with reversal potential 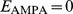 mV. For the inhibitory GABAA synapses, the maximum conductances were 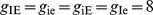 pS/μm^2^ and 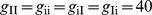 pS/μm^2^, with reversal potential 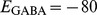 mV.

Oscillation frequency is influenced by the decay time constant of the inhibitory GABAA conductance (i.e., the IPSC decay constant, 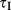) [2], [26], [40]. We chose 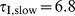 ms for the slow network and 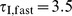 ms for the fast network. In combination with the strength of the maximal GABAA conductance, which was the same in the slow and the fast network (see above), this resulted in an oscillation frequency of about 20.4 Hz for the slow network in isolation and a frequency of about 32.4 Hz for the fast network in isolation. The IPSC decay constant of the inhibitory connections from the source to the target network was taken the same as the IPSC decay constant of the inhibitory connections within the source network. The decay time constant of the AMPA conductance was 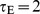 ms. The synaptic delay for both excitatory and inhibitory synapses was 1 ms [46].

### External drive

Each excitatory and inhibitory cell in both the slow and the fast network received a constant depolarizing current 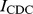 [8], representing tonic cholinergic input necessary to induce the oscillations [12], [47] that are generated by the reciprocal interactions between the E and I cells.

Cholinergic input has been shown to cause a sustained depolarizing response [48] that, as we do here, can be mimicked by applying a non-specific, depolarizing current to the cells [12]. The amplitude of 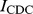 varied among cells and was randomly drawn from a uniform distribution in the intervals [3.8–6.3] pA for the inhibitory population in both networks and [10.1–11.3] pA for the excitatory population in both networks. The values of the currents were based on results from [8], [49] and were fixed for the duration of the stimulation.

### Connectivity schemes between networks

**Figure 2:**
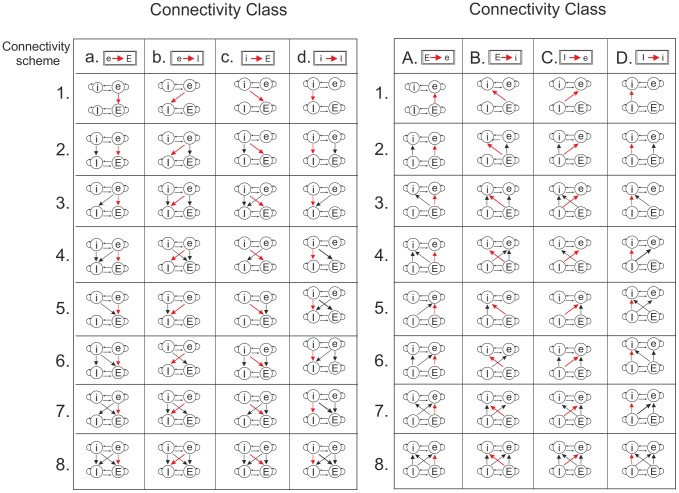
The connectivity schemes between the two model networks. In the slow network, the excitatory and inhibitory populations are labelled with lower case letters (e, i) and in the fast network with upper case letters (E, I). Each column (a–d, A–D) comprises what we call a connectivity class, consisting of eight different connectivity schemes. The strength of the connectivity type shown in red was varied in the simulations. A connectivity class is labelled with a lower or upper case letter depending on whether the slow or the fast network, respectively, is the network projecting to the other network. (See further Methods.)

We considered all possible feed-forward connectivity schemes between the two networks (Fig. 2). The left 4×8 block of Fig. 2 shows the connectivity schemes in which the slow network (cells labelled by lower case letters: e, i) projects to the fast network (upper case letters: E, I). The right 4×8 block shows the schemes in which the fast network projects to the slow network. In each connectivity scheme, the synaptic strength of one type of connection (shown in red) was varied systematically (see below), whereas the strengths of all other connection types were kept fixed. Each column in Fig. 2 comprises what we call a connectivity class, in which the connection type whose synaptic strength was varied is considered in eight different connectivity schemes of the fixed connections. A connectivity class is also labelled with a lower or upper case letter depending on whether the slow or the fast network, respectively, is the source network. Recall that, because the connectivity from the source to the target network was created by connection probabilities, not all target cells may receive connections from the source network. The set of connected cells, however, was the same for all connectivity schemes.

In each of the 64 connectivity schemes, we considered ten different synaptic strengths (conductances) of the variable connection, obtained by multiplying the default strength by a conductance factor Cf  =  [0.01, 0.05, 0.5, 0.8, 1.5, 3.0, 7.0, 10.0, 15.0, 20.0]. In each of these 10×64 = 640 simulation conditions, we recorded the activity of all cells in both networks for 40 s.

### Analysing network dynamics

Network dynamics was analysed separately for the four populations in the model: the e and the i population of the slow network, and the E and the I population of the fast network. We analysed the dynamics by means of firing-rate histograms, Fourier analysis and wavelet analysis. Because the activity profiles of the excitatory and the inhibitory population turned out to be very similar, we show only results from the excitatory populations.

### Firing-rate histograms

To describe the network activity in each of the populations, we constructed firing-rate histograms by counting spikes in time bins of 6 ms. This bin size relates to a sample frequency of about 167 Hz, much higher than the oscillation frequencies in our simulations. Because this bin size practically eliminated the occurrence of more than one spike per time bin per cell, the number of spikes was equal to the number of active cells per time bin. Whilst the source network produced a robust and unperturbed oscillation, the activity in the target network was the outcome of the interaction between its own oscillation and oscillatory input from the source network.

### Fourier analysis

The fast Fourier transform of the activity in each cell population (four cell populations: the excitatory and inhibitory populations in the target and source networks) was computed on the basis of the firing-rate histograms (of 40 s of activity). To smoothen the histograms, we first convolved them with an alpha function 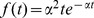, where 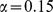 and *t* is in 6 ms time units (the bin size); 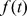
was evaluated for five consecutive time bins. The convolved signal was used as input for the Welch's periodogram Matlab algorithm. For each cell population, we identified the components in the fast Fourier transform that were local maxima; the maximum of these components is then the peak frequency of that particular cell population. In the figures, the oscillation frequencies in the target network, as revealed by the Fourier analysis, are indicated by blue discs. The diameters of the blue discs indicate the power of the frequency components in the excitatory population of the target network; only frequency components are shown whose power was larger than 30% of the peak power in the excitatory population of the source network. The oscillation frequencies in the source network are indicated by red dots (of fixed size), which show the location of the base frequency and the first harmonic, but not their power. The green arrow points to the base frequency of the source network.

### Wavelet analysis

To analyse how the power (amplitude) of the oscillations varied over time, we performed a wavelet analysis using the Torrence algorithm [50] implemented in MatLab, with the convolved firing-rate histogram as input. A standard Morlet function was used with a frequency range of 0.01–70 Hz and 0.1 Hz scaling windows. The y-axis of the wavelet plots, in most cases running from 0 to 50 Hz, was chosen so as to depict the frequencies around the frequencies of the slow and fast networks (20.4 and 32.4 Hz, respectively).

### Some definitions

The base frequency of a network oscillation is defined as the number of periodic states of synchronous cell firing in one second (i.e., the conventional physical definition). The first harmonic of the oscillation, as visualized in the Fourier spectrum, corresponds to a firing pattern in which the synchronous states occur at a frequency of twice the base frequency. By induction, the *n* ^th^ harmonic frequency is defined as 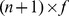, where *n*>0 is an integer and *f* is the base frequency. Likewise, a subharmonic frequency is defined as a frequency that can be written as 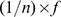, where *n*>1 is an integer.

## Results

**Figure 3:**
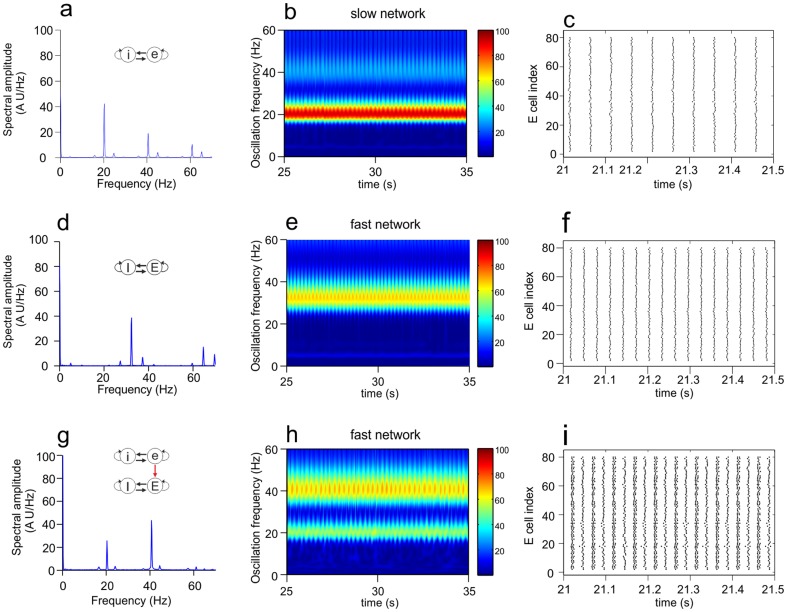
Oscillatory activity in the slow and the fast network when they are unconnected or connected. Shown are the Fourier transform (**a, d, g**), wavelet transform (**b, e, h**) and raster diagram of cell firing (**c, f, i**) of the excitatory population in either the slow or the fast network. Color in the wavelet transforms indicates power of oscillation. The raster diagrams depict the firing times (indicated by dots). **a–c.** Activity of the slow network in isolation. The Fourier transform shows peaks at the base frequency (20.4 Hz) and at the first and second harmonics. Owing to the highly synchronized activity (making the signal effectively a comb function), the Fourier transform produced peaks at the harmonics, but there were no cells that actually fired at these frequencies (see panel c). **d–f**. Activity of the fast network in isolation. The Fourier transform shows peaks at the base frequency (32.4 Hz) and the first harmonic. **g–i.** Activity of the fast network when the excitatory cells of the slow network projected to the excitatory cells of the fast network (eE connection) with conductance factor 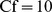 (see Methods). With this connection strength, the slow network managed to impose its rhythm onto the fast network, in which the base frequency (20.4 Hz) of the slow network and its first harmonic were strongly expressed. Since there were no connections from the fast to the slow network, the activity of the slow network was not different from that in the unconnected situation.

We considered two networks and investigated how feed-forward connections from one network (the source network) to the other network (the target network) affected the activity in the latter. In Fig. 3, we first demonstrate that each network in isolation generated a stable oscillation at a given frequency. Because the networks had different IPSC decay constants, one network had an oscillation frequency of 20.4 Hz (the slow network; Figs. 3a–c) and the other one a frequency of 32.4 Hz (the fast network; Figs. 3d–f). The oscillations were caused by the interactions between the excitatory and inhibitory cells and were driven by the constant depolarizing currents (CDCs) provided to all the cells (see Fig. 1c and Methods).

Fig. 3 also shows that the activity of one network could markedly influence the activity in the other network when the networks were connected (Figs. 3g–i). In this example, the excitatory cells of the slow network (in this case, the source network) projected to the excitatory cells of the fast network (the target network) with synapses that were 10 times stronger than the excitatory synapses within each network (i.e., the conductance factor 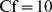; see Methods). With this connection strength, the slow network completely overruled the rhythm of the fast network. The fast network could no longer express its own rhythm and displayed only the base frequency and first harmonic of the slow network.

To investigate systematically how the influence of the source network on the oscillatory activity in the target network depended on the pattern and strength of the connections from source to target network, we considered all possible feed-forward connectivity schemes between the two networks (Fig. 2). For each connectivity scheme, we varied the strength of one connection type, while the strengths of all other connection types were kept fixed.

### Impact of source network on oscillation frequency in target network

As will be described in more detail below, we found that for high inter-network connection strengths, the source network could completely impose its rhythm onto the target network. Interestingly, the slow network was in general more effective at imposing its rhythm onto the fast network than the other way around. For lower inter-network connection strengths, multiple oscillation frequencies, i.e., the own frequency of the target network and the own frequency of the source network, could coexist in the target network, especially when the slow network acted as source network.

### The slow network imparts its rhythm onto the fast network especially when it has strong excitatory connections to the excitatory or inhibitory cells of the fast network

We first investigated the impact of the oscillatory activity of the slow network on the oscillatory activity of the fast network. Thus, the slow network acted as the source network and the fast network as the target network.

**Figure 4:**
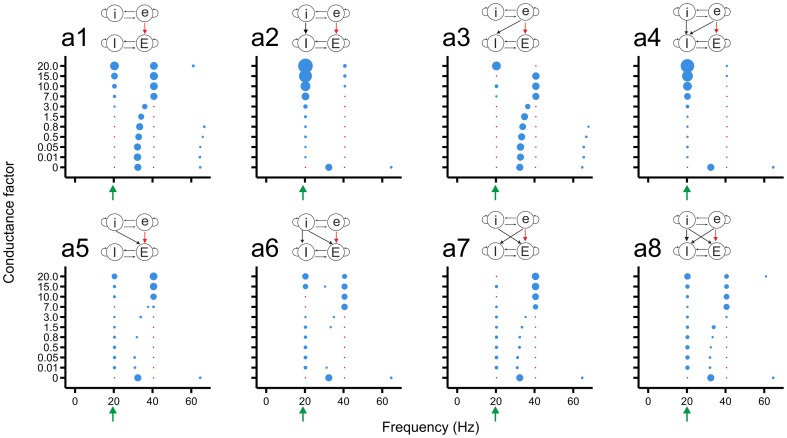
The eE connections from the slow to the fast network can impose the slow rhythm onto the fast network already at low connection strengths. Lower case letters (e, i) label the excitatory and inhibitory populations in the slow network, and upper case letters those in the fast network (E, I). The strength (conductance factor) of the eE connections (red arrow in connection scheme) is varied in the different connectivity schemes. The blue discs indicate the oscillation frequencies in the fast network; their diameters depict the power. The red dots show the base frequency and the first harmonic of the slow network, without indicating power. The green arrow points to the base frequency of the slow network. In all connectivity schemes, for high eE connection strengths, the fast network became frequency locked to the rhythm of the slow network, at its base frequency and/or at the corresponding first harmonic. For lower connection strengths, the base frequency of the slow network and a frequency close to the base frequency of the fast network coexisted in the fast network (e.g., a7, a8).

In connectivity class a (Fig. 4), in which the strength of the eE connections was varied, the peak frequency of the fast network gradually shifted to the first harmonic of the slow network for increasing eE connection strength (Fig. 4a1). For connection strengths 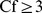, also the base frequency of the slow network appeared in the fast network. In the presence of iI connections (Fig. 4a2), the base frequency of the slow network already appeared for low connection strength. With eI connections also included (Fig. 4a3), the frequency of the fast network gradually shifted to the first harmonic of the slow network, but only for the highest eE connection strength did it become locked to the base frequency of the slow network. With additional iE connections (Figs. 4a5–a8), the fast network slowly moved to the first harmonic of the slow network as the eE connection strength increased, while the base frequency of the slow network was already present for low connection strengths. For lower eE connection strengths and in the presence of iE connections, the base frequency of the slow network and a frequency close to the base frequency of the fast network could coexist (Figs. 4a5, a7, a8).

**Figure 5:**
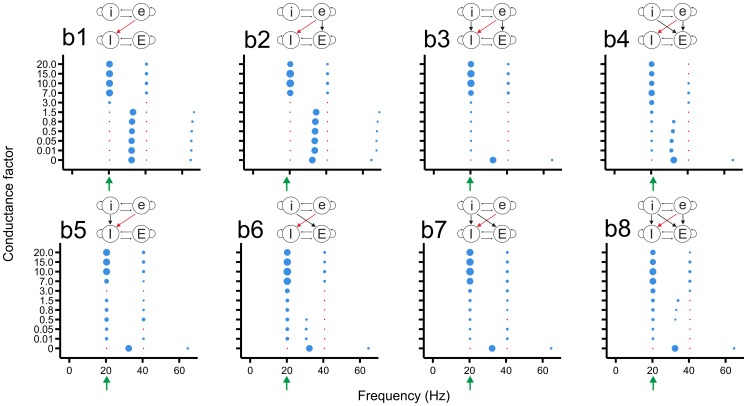
The eI connections from the slow to the fast network can impose the slow rhythm onto the fast network already at low connection strengths. In all connectivity schemes, the fast network oscillated at the base frequency of the slow network (and its first harmonic) for high eI connection strength. For lower strengths, two different frequencies could coexist (b4, b6, b8).

In connectivity class b (Fig. 5), in which the strength of the eI connections was varied, the fast network initially maintained its base frequency for low eI connection strengths (Fig. 5b1). For increasing strength (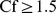), the fast network jumped to the base frequency of the slow network and got entrained into this frequency and its first harmonic. Adding eE connections (Fig. 5b2) did not change this pattern. In the other connectivity schemes (Figs. 5b3–b8), the base frequency of the slow network already appeared for lower eI connection strengths. In some connectivity schemes (Figs. 5b4, b6, b8), the base frequency of the slow network and a frequency close to the base frequency of the fast network could coexist for low or intermediate connection strengths.

In the other two connectivity classes from the slow to the fast network (classes c and d; Figs. S1 and S2), the slow network could also impart its rhythm onto the fast network, although less effectively. Moderate to high iE connection strength brought the base frequency of the slow network into the fast network (Fig. S1), but generally with lower power than for connectivity classes a and b. In addition, a frequency component close to the base frequency of the fast network could remain in the fast network even for high connection strengths (Figs. S1c4, c5). Also high iI connection strengths forced the fast network to oscillate at the slow base frequency and/or its first harmonic (Fig. S2), but again with lower power than for connectivity classes a and b. In some cases, the fast network was still able to additionally express its own base frequency (Figs. S2d7, d8).

In conclusion, the strongest entrainment of the rhythm of the fast network to that of the slow network occurred when excitatory cells of the slow network projected to excitatory or inhibitory cells of the fast network (Figs. 4 and 5, respectively). Both the slow base frequency and its first harmonic could appear in the fast network. Locking of the rhythm in the fast network to the frequency of the slow network was also possible when inhibitory cells of the slow network projected to excitatory or inhibitory cells of the fast network (Figs. S1 and S2, respectively), although the power was generally low. In many connectivity schemes, especially for low connection strengths, the fast network could maintain its own frequency and at the same time express the frequency of the slow network.

### Mechanism underlying the entrainment of the fast network to the rhythm of the slow network

**Figure 6:**
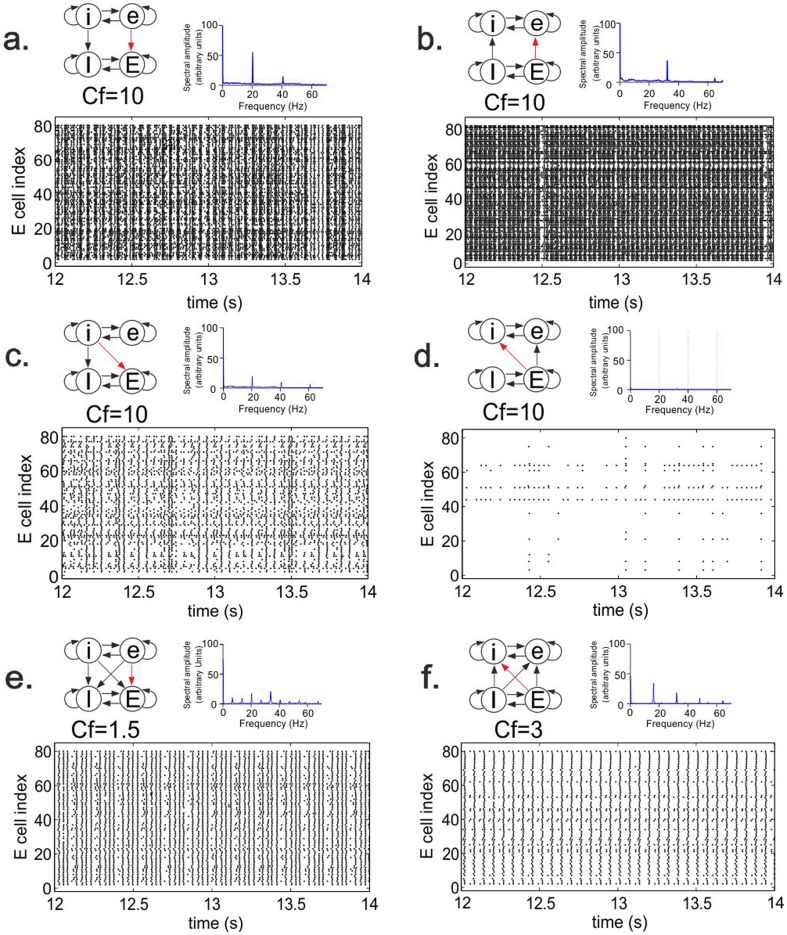
The effect of inter-network connections on firing pattern. Shown are, for different connectivity schemes, the Fourier transform and the raster diagram of cell firing in the excitatory population of the target network. The conductance factor Cf indicates the relative strength of the red connection (see Methods). Panels **a–f** show connectivity schemes a2, A2, c2, B2, a8 and B8, respectively (see Fig. 2). In **a–d**, the source network completely imposed its rhythm onto the target network, whereas in **e** and **f** two different non-harmonic frequencies could coexist. In **c** and **d**, increased inhibition in the target network due to strong iE or Ei connections reduced the power of oscillatory activity in the target network.

As mentioned, the slow network could impart its rhythm onto the fast network when it had strong excitatory connections to the excitatory cells of the fast network (Figs. 4, 6a). Excitatory cells from the slow network provide strong excitation to the excitatory population of the fast network, recruiting excitatory cells to fire at the rhythm of the slow network and, via connections from excitatory to inhibitory cells within the fast network, also enhancing the activity in the inhibitory population at a frequency of the slow rhythm, which then in turn inhibits the excitatory population.

The entrainment of the fast network to the rhythm of the slow network with strong excitatory connections from the slow network directly onto the inhibitory cells of the fast network (Fig. 5) occurs through a similar mechanism but then without the intervention of the fast network's excitatory population. Compared with excitatory inter-network connections, strong inhibitory connections from the slow to the fast network (Figs. S1, 6c) imposed the rhythm of the slow network with lower power, mainly because inhibition also reduces the overall activity in the fast network (Fig. 6c). In addition, there are fewer inhibitory than excitatory inter-network connections because the inhibitory population is smaller than the excitatory population.

For lower inter-network connection strengths, the base frequencies of the slow and the fast network could coexist in the fast network. In Fig. 6e, for example, both frequencies were expressed in an alternating manner. This might reflect some sort of interference, since each time after a cluster of four synchronized firing states, with within-cluster intervals of around 30 ms (the fast frequency), the next cluster occurred with an interval of around 50 ms (the slow frequency).

### The fast network imparts its rhythm onto the slow network especially when it has strong excitatory connections to the excitatory cells of the slow network

Next, we studied the impact of the oscillatory activity of the fast network on that of the slow network. Thus, now the fast network acted as the source network and the slow network as the target network.

**Figure 7:**
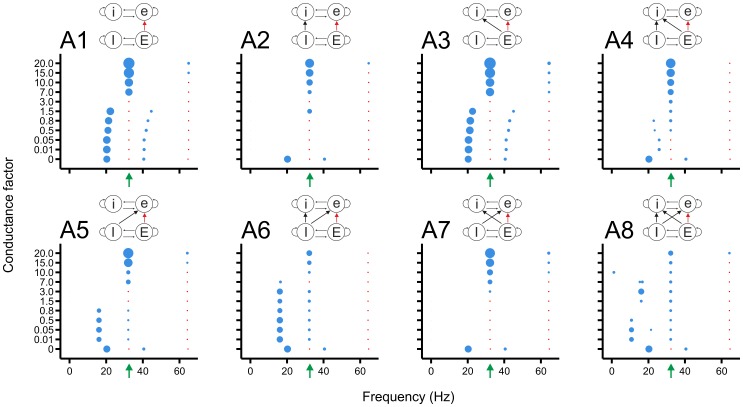
The Ee connections from the fast to the slow network can impose the fast rhythm onto the slow network, especially at high connection strengths. The blue discs now indicate the oscillation frequencies in the slow network; their diameter depicts the power. The red dots show the base frequency and the first harmonic of the oscillatory activity in the fast network, without indicating power. The green arrow points to the base frequency of the fast network. For high Ee connection strength, the slow network became fully entrained to the rhythm of the fast network. For intermediate Ee connection strengths, some connectivity schemes (e.g., A6) exhibited an activity pattern in which two different oscillation frequencies coexisted.

In connectivity class A (Fig. 7), in which the strength of the Ee connections was varied, the peak frequency of the slow network moved quite abruptly to the base frequency of the fast network for 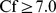 (Fig. 7A1). When also Ii connections were included (Fig. 7A2), the power in the slow network became very low as the Ee connection strength increased until the connection strength was 1.5. The other transition patterns were the same (Fig. 7A3) or more complex (Figs. 7A4–A8) than in Fig. 7A1. In Fig. 7A6, for a large range of Ee connection strengths, the base frequency of the fast network coexisted with a frequency that was slightly lower than the base frequency of the slow network. In Fig. 7A7, as in Fig. 7A2, the power in the slow network became very low for low and intermediate Ee connection strengths.

In connectivity classes B and C (Figs. S3 and S4), in which respectively the Ei and Ie connection strengths were varied, the fast network was generally not able to impose its rhythm onto the slow network, and in many cases strongly inhibited the oscillatory activity in the slow network. Even for low Ei connection strengths, the fast network could strongly reduce the oscillation power in the slow network (Fig. S3). In all connectivity schemes of class B, the fast network was not able to impose its rhythm onto the slow network for high Ei connections strengths. Only in some connectivity schemes did entrainment to the fast network occur for relatively low connection strengths (Figs. S3B3, B8). In one connectivity scheme (Fig. S3B8), the base frequency of the fast network could coexist with a frequency that was slightly lower than the base frequency of the slow network. Also in connectivity class C, the fast network strongly reduced the oscillation power in the slow network (Fig. S4), even for low Ie connection strengths, and was hardly able to impose its rhythm onto the slow network. Two different frequencies could occasionally coexist (Figs. S4C6, C8).

In connectivity class D (Fig. S5), in which the strength of the Ii connections was varied, the fast network could in most connectivity schemes shift the oscillation frequency in the slow network to the fast base frequency for high Ii connection strength, albeit with varying power. In some connectivity schemes, the base frequency of the fast network could coexist with a frequency that was lower than the base frequency of the slow network (Figs. S5D7, D8).

In conclusion, entrainment of the rhythm in the slow network to the frequency of the fast network was observed only when excitatory cells of the fast network projected to excitatory cells of the slow network (Fig. 7) or when inhibitory cells of the fast network projected to inhibitory cells of the slow network (Fig. S5), although in the latter case with low power. When no entrainment occurred, the main effect of the fast network was that it strongly suppressed the oscillatory activity in the slow network. For low connection strengths, patterns with multiple frequencies, in which the slow network simultaneously expressed its own frequency and the frequency of the fast network, also occurred, but less extensively than when the slow network projected to the fast network.

### Mechanism underlying the entrainment of the slow network to the rhythm of the fast network

Why is the fast network in general less capable of implanting its rhythm on the slow network than the other way around? In order for the fast network to impose its rhythm onto the slow network, it must induce the cells in the slow network to fire at a higher frequency, which requires strong enough excitation both to overcome the extensive hyperpolarization due to the long IPSC and to exceed the firing threshold. In contrast, in order for the slow network to impose its rhythm onto the fast network, any extra inhibition, either directly via excitatory input from the slow network to the inhibitory cells of the fast network (Fig. 5) or indirectly via excitatory input from the slow network to the excitatory cells of the fast network (Fig. 4), can already diminish the firing frequency in the fast network. In other words, it is easier to inhibit a depolarized cell than to cause a hyperpolarized cell to fire.

The fast network could impose its rhythm onto the slow network when it had strong excitatory connections to the excitatory cells of the slow network (Fig. 7, 6b). Strong excitatory connections from the fast network directly onto the inhibitory cells of the slow network (Fig. S3), however, increased the amount of inhibition, thereby strongly reducing the oscillatory activity in the slow network (Fig. 6d). For lower inter-network connection strengths, two different non-harmonic frequencies could coexist in the slow network. In Fig. 6f, for example, a fraction of the cells constantly fired at a higher frequency.

### Impact of source network on temporal oscillation patterns in target network

In the previous sections, we showed that the source network could change the frequency spectrum in the target network, but not whether frequency and amplitude might also vary over time. Using wavelet analysis, we found that the source network could induce a large variety of temporal patterns of oscillatory activity in the target network, ranging from patterns in which multiple frequencies were almost continually expressed to those in which the co-expressed frequencies appeared intermittently in time or in which single or multiple frequencies fluctuated in amplitude. The examples described in the next sections are representative of the different types of temporal patterns observed in the target network.

### Continual or alternating expression of two non-harmonic oscillation frequencies

**Figure 8:**
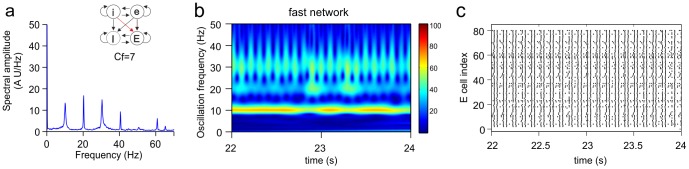
Continual expression of coexistent oscillation frequencies. Shown are the Fourier transform (**a**), wavelet transform (**b**) and raster diagram of cell firing (**c**) from the excitatory population of the target network (the fast network). The inset in **a** shows the connectivity scheme from the slow to the fast network (see Fig. S1c8), in which the iE connection had 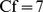. The fast network co-expressed its own fast base frequency (32.4 Hz) and the base frequency of the slow network (20.4 Hz and corresponding harmonic and subharmonic frequencies). The power (amplitude) of both frequencies did not vary strongly over time.

Fig. 8 shows a situation in which two different frequencies, the base frequency of the slow network (source network) and the base frequency of the fast network (target network), occurred almost concurrently in the fast network. In addition to the base frequencies, the fast network continually expressed a subharmonic of the slow base frequency.

**Figure 9:**
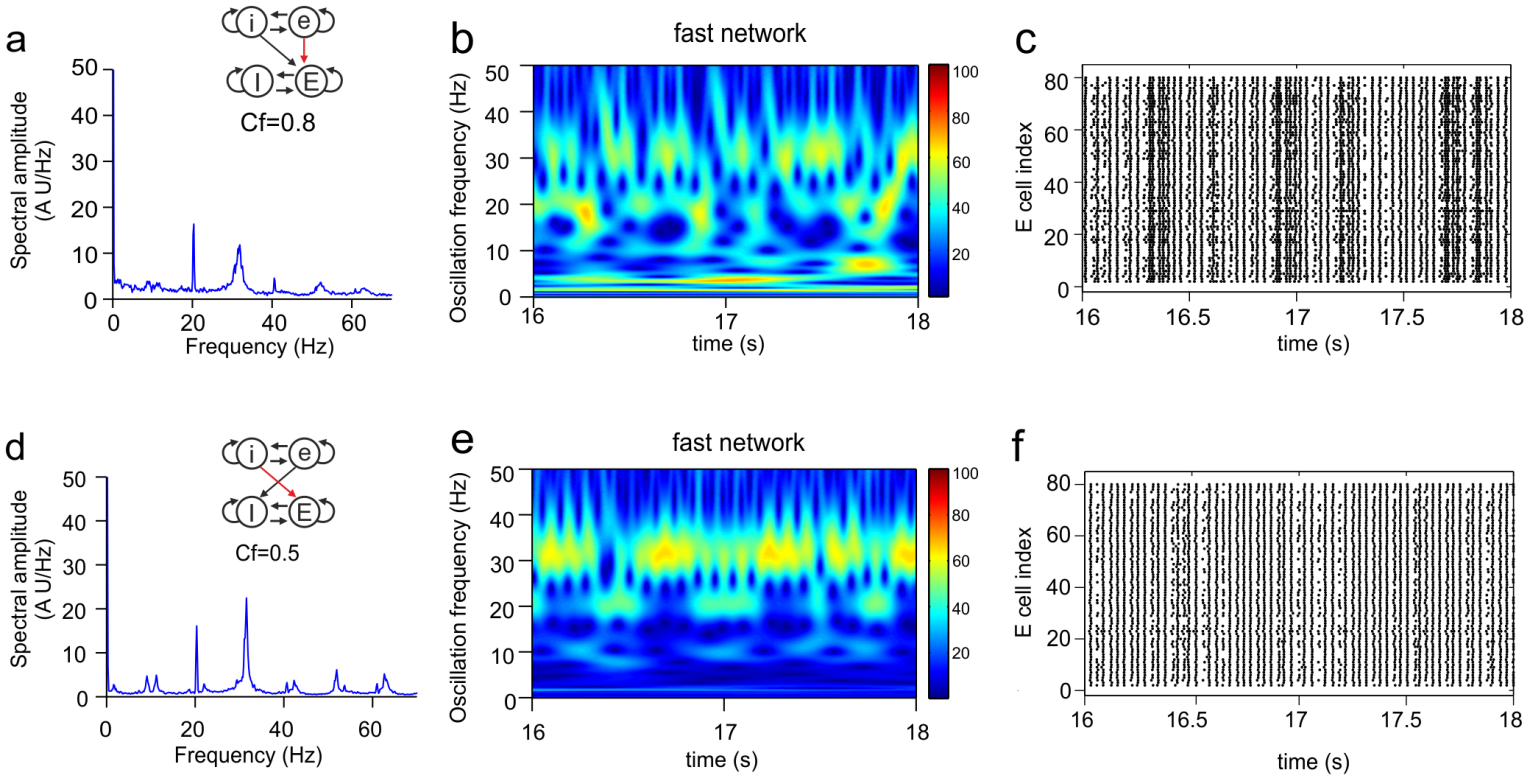
Alternating expression of coexistent oscillation frequencies. Shown are the Fourier transform (**a, d**), wavelet transform (**b, e**) and raster diagram of cell firing (**c, f**) from the excitatory population of the target network (the fast network). The insets in **a** and **d** show the connectivity schemes from the slow to the fast network (see Figs. 4a5 and S1c4, respectively), in which the eE connection (**a**) had 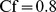 and the iE connection (**d**) had 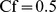. For both connectivity schemes, the base frequency of the fast network (32.4 Hz) and the base frequency of the slow network (20.4 Hz) appeared intermittently in the fast network. In **b**, the frequency switched faster than in **e**. When either frequency component was present, its power remained relatively constant.

Fig. 9 shows two cases in which also two different frequencies were expressed in the fast network, the base frequency of the slow network and the base frequency of the fast network, but in which the frequencies clearly appeared in an alternating manner. When one frequency had high power, the other frequency was strongly reduced in power or was absent. The episodes in which either of the two frequencies was the dominant frequency were longer in Fig. 9e than in Fig. 9b. Occasionally, both frequencies occurred almost simultaneously (e.g., around t = 16.1 and 16.5 s in Fig. 9b) with low power. The alternating expression of the two frequencies results from interference between the two frequencies. The cells in the fast network continually sense both the base frequency of the fast network and the base frequency of the slow network, and alternately lock to either the fast or the slow rhythm.

### Temporal fluctuations in oscillation power

**Figure 10:**
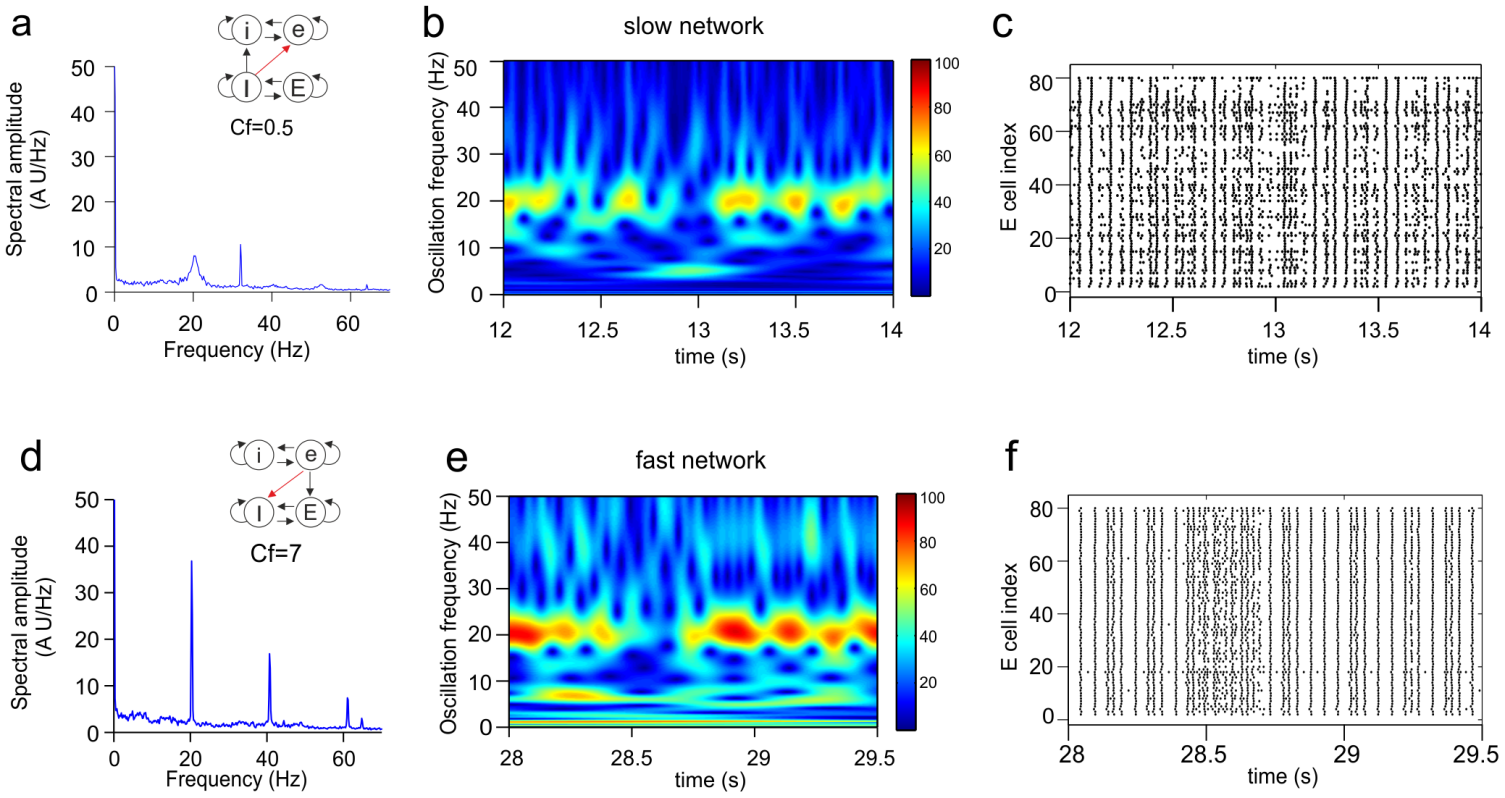
Expression of a single oscillation frequency with strong fluctuations in power. Shown are the Fourier transform (**a, d**), wavelet transform (**b, e**) and raster diagram of cell firing (**c, f**) from the excitatory population of the target network (the slow network in **a–c** and the fast network in **d–f**). The inset in **a** shows the connectivity scheme from the fast to the slow network (see Fig. S4C2), in which the Ie connection had 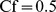. In the time interval shown, the slow network expressed its own base frequency (20.4 Hz) but with strong fluctuations in power. The inset in **d** shows the connectivity scheme from the slow to the fast network (see Fig. 5b2), in which the eI connection had 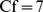. The fast network expressed the base frequency of the slow network with strong fluctuations in power.

Fig. 10 shows two cases in which only one main frequency was present in the target network but with varying power over time. In the first case (Figs. 10a–c) the slow network was the target network, and in the second case (Figs. 10d–f) the fast network acted as target. In both cases, the power of the frequency in the target network fluctuated irregularly, with episodes of high power alternating with episodes of low power (Figs. 10b, e). The amplitude or power of a particular oscillation frequency is determined by the number of synchronously firing cells at that frequency. As can be seen by comparing Figs. 10e and f, episodes of low power in the wavelet (e.g., around t = 28.5 s) correspond to episodes of desynchronized firing.

**Figure 11:**
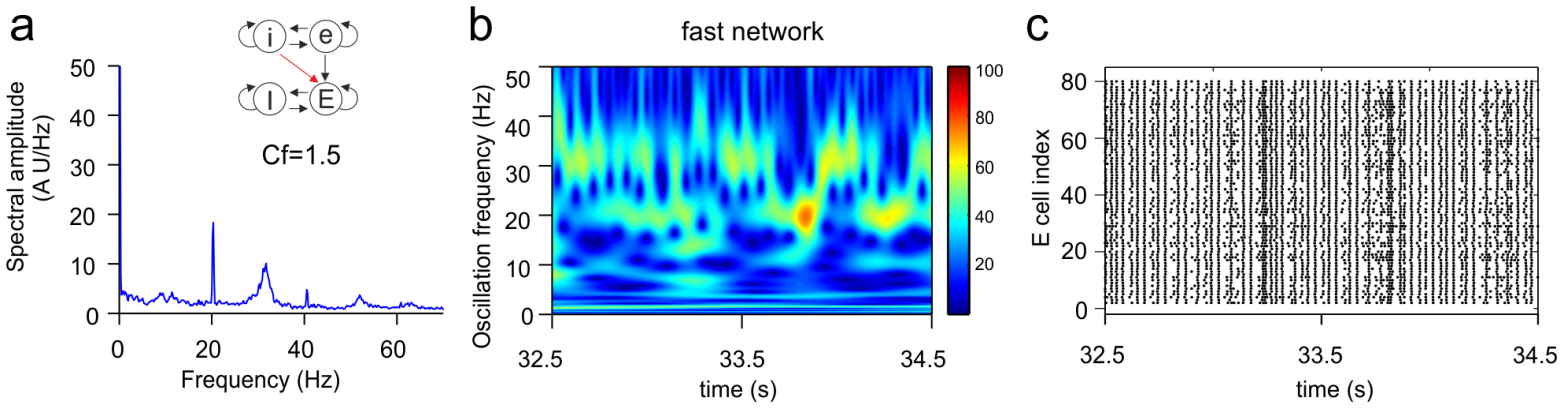
Alternating expression of coexistent oscillation frequencies with fluctuations in power. Shown are the Fourier transform (**a**), wavelet transform (**b**) and raster diagram of cell firing (**c**) from the excitatory population of the target network (the fast network). The inset in **a** shows the connectivity scheme from the slow to the fast network (see Fig. S1c5), in which the iE connection had 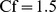. The base frequency of the fast network (32.4 Hz) and the base frequency of the slow network (20.4 Hz) appeared more or less intermittently in the fast network. When either frequency component was present, its power was not stable over time (e.g., the slow base frequency between t = 33.7 s and t = 33.9 s).

Fig. 11 shows a situation in which again two frequencies, the base frequency of the slow network (source network) and the base frequency of the fast network (target network), coexisted in an alternating fashion in the fast network. However, more so than in the cases shown in Fig. 9, each frequency also fluctuated in power during the episodes that it was the dominant frequency. For example, from about t = 33.7ϖs to t = 33.9 s, the base frequency of the slow network was the only frequency component in the network, but its power was not constant over time (Fig. 11b).

What is the cause of fluctuations in oscillation power? In a previous study [51], we have shown that in a single network, action potentials from areas external to the network and impinging onto the inhibitory cells can disrupt synchronous firing. Since oscillation amplitude is proportional to the number of simultaneously firing cells, reduced synchrony gives rise to a decrease in oscillation amplitude (power). This desynchronization effect of the external action potential input competes with the tendency of the interacting excitatory and inhibitory cells to drive the network back to synchrony, causing alternating episodes of high-amplitude oscillations (high power) and low-amplitude oscillations (low power). In the two-network model studied here, the external action potential input is provided by the source network, which can likewise induce amplitude fluctuations. Amplitude fluctuations in ongoing oscillations are ubiquitous and have been observed in the intact brain as well as in cortical slices for many frequency bands [27], [28], [52].

### Comparison with experimental data on alternating expression of oscillation frequencies

**Figure 12:**
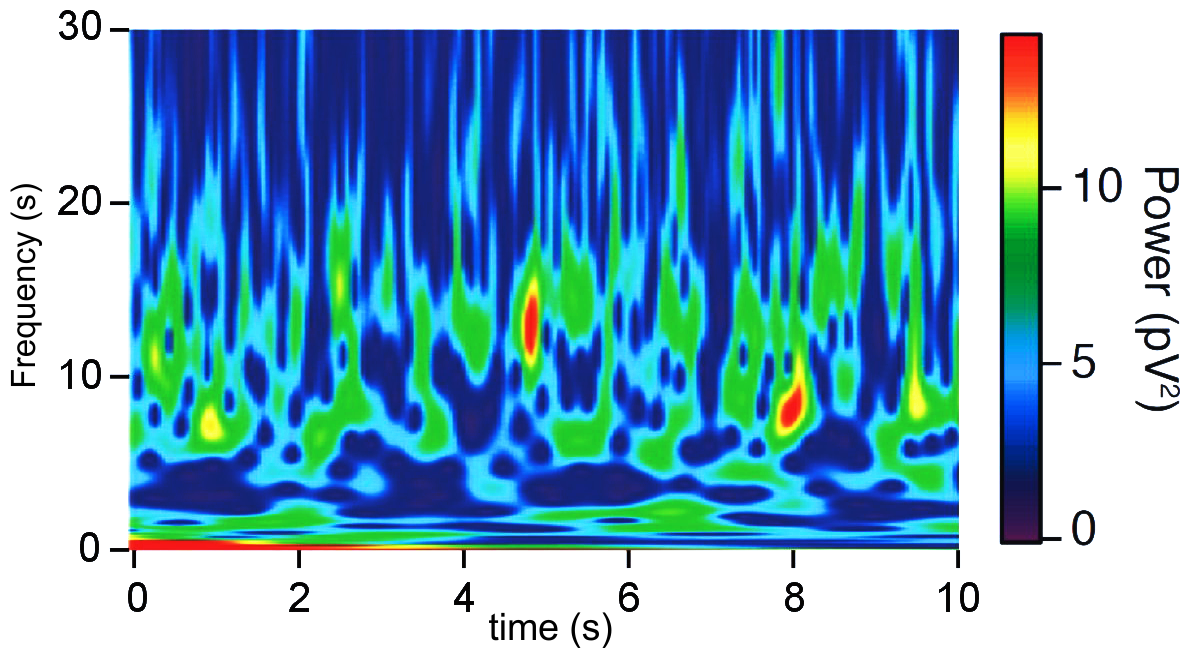
Alternating expression of two coexistent oscillation frequencies in rat PFC. Wavelet transform of extracellular field potential in layer 5 of rat PFC slice. Two different non-harmonic frequencies, around 7 Hz and 13 Hz, alternate as dominant frequency but are occasionally expressed simultaneously (e.g., around t = 5.5 s). The figure was not published before but is based on data collected in [26].

Our results suggest that interacting oscillatory networks could, at least partially, be responsible for the rich repertoire of oscillation patterns observed in the brain. Fig. 1 reveals distinct oscillation frequencies appearing intermittently in time in the human brain (Fig. 1a) and rat prefrontal cortex (Fig. 1b). Another example of alternating frequencies in rat PFC is given in Fig. 12, which shows similar dynamics to that generated by our model networks in Figs. 9b and 11b. In Fig. 12, the two frequencies mostly alternate, but occasionally occur simultaneously (e.g., around t = 5.5 s). In the PFC, fast oscillations are primarily found in layer 3/5, whereas slow oscillations occur predominantly in layer 6 [26]. In cortical layer 5, episodes of both slow and fast oscillations are present. The experimental data suggest the presence of two local feedback networks located in layer 3 and layer 6 that each oscillate at their own frequency [26]. The interactions between these two networks may lead to the alternating expression of frequencies observed in layer 5.

## Discussion

Brain networks capable of generating oscillations are typically strongly connected to each other [23], but the impact of these connections on oscillatory activity is not well known [32]. We created two model networks, each producing a different oscillation frequency on its own, and investigated systematically the influence of inter-network connections on oscillation frequency and pattern. Either the slowly oscillating network projected to the fast oscillating network (i.e., the slow network acted as source network and the fast network as target network), or the fast oscillating network projected to the slowly oscillating network.

For high inter-network connection strengths, the source network could completely impose its rhythm on the target network. In general, the slow network was better able to impart its rhythm onto the fast network than the other way around. The strongest locking of the oscillation frequency in the fast network to the frequency of the slow network occurred when excitatory cells of the slow network projected to excitatory or inhibitory cells of the fast network. The fast network could most strongly impose its rhythm on the slow network when its excitatory cells projected to the excitatory cells of the slow network. In situations where no entrainment to the fast frequency took place, the fast network greatly suppressed the oscillatory activity in the slow network.

For lower inter-network connection strengths, multiple oscillation frequencies, i.e., the own frequency of the target network and the own frequency of the source network, could coexist in the target network, especially when the slow network acted as source network. Interestingly, the target network exhibited a wide range of temporal patterns: the target network could express multiple frequencies at the same time, alternate between distinct oscillation frequencies, or express only a single frequency but with alternating episodes of low and high power. Intermediate patterns were also possible, in which two frequencies in turn were the dominant frequency and each frequency fluctuated in power when it was dominant.

In general, networks that generate oscillations by means of interacting excitatory and inhibitory cells (PING mechanism) have a tendency to maintain oscillation frequency even in the face of small perturbations [40]. In the two-network constellation, the input from the source network delays neuronal firing (when the slow network is the source network) or advances neuronal firing (when the fast network is the source network) in the target network. The effect of this disruption competes with the tendency of the target network to maintain its own base frequency. With strong connections from the source to the target network, the oscillating input from the source network overrules the base frequency of the target network, and the target network will oscillate at the frequency of the source network. With weak inter-network connections, the cells in the target network continually sense both the base frequency of the source network and the base frequency of the target network, and can alternately lock to either frequency. To obtain a better theoretical understanding of network entrainment and coexistence of frequencies, one could analyze the phase delays and advances in firing that occur in the target network as a result of the perturbations due to the input from the source network (see also [51]).

The choice of oscillation frequencies for the fast and the slowly oscillating network was based on frequencies reported for the hippocampus [41], [42], [43] and the PFC [3], [26], as well as on the difference in oscillation frequency observed between layer 3/5 and layer 6 of rat PFC [3], [26]. Although we did not systematically analyse other oscillation frequencies, preliminary tests indicate that our main results are not critically dependent on the precise choice of frequencies as long as the difference in oscillation frequency between the two networks is not too large. Relevant in the choice of oscillation frequencies is whether the source network can interfere with the target network, and whether the target network can recover from a perturbation caused by the source network. If the target network (high frequency) rapidly recovers (i.e., resumes its own rhythm) before the next perturbation from the source network (low frequency) arrives, one may not expect any ongoing interference between rhythms. For instance, in a test case it took about 3–4 oscillation cycles in the fast network to recover from a perturbation caused by the source network. In such a situation, the source frequency should not be lower than about four times the target frequency.

We did not consider reciprocal connections between the slow and the fast network. In the connectivity schemes we analysed, either the slow network projected connections to the fast network or the fast network projected connections to the slow network. Already with this restriction, the number of possible connectivity schemes (Fig. 2), together with the number of different connection strengths tested, leads to a very large number of simulation conditions. Pilot experiments indicated that with reciprocal connections, whichever connections were the strongest, those from the slow to the fast network or from the fast to the slow network, were the dominant connections. Thus, also with reciprocal connections, the uni-directional connectivity schemes may be useful in predicting the effect of connection type and strength on oscillatory activity.

Each of the two networks was composed of 80 excitatory cells and 20 inhibitory cells. In our previous work [51], in which we considered only a single network but with the same composition as the networks used here, we found that increasing the total number of cells in the network ten-fold, while maintaining the values of all other parameters and the proportion of excitatory and inhibitory cells, did not affect the results. Although network size may have an effect on oscillation frequency [53], we do not expect that our conclusions critically depend on network size.

The connection strength from the source to the target network was varied by changing the excitatory or inhibitory synaptic conductance. When an excitatory or inhibitory population of the source network was connected to an excitatory or inhibitory population of the target network, not all the cells of the respective populations were connected, but only a fraction (see Methods). Fig. S6 illustrates that this fraction also had an impact on the ability of the source network to influence the dynamics in the target network (see further Text S1).

Our study may stand as a useful reference point for future computational studies using more complex neurons (e.g., dendritic morphology, ion channel composition), different network constellations (e.g., reciprocal connectivity) or other oscillation mechanisms (e.g., interneuron network gamma, or ING, without direct involvement of excitatory cells; [40]). Although altering the neurons or networks could potentially modify some of our detailed findings, we expect that the mechanisms by which oscillations influence each other in our simulated networks are quite robust—being dependent on the interference of different rhythms and the interaction between neurons with different IPSC decay constants—and would also play a role in more complex neuronal or network implementations.

Relatively few model studies have considered interacting oscillatory networks. In a model consisting of two layer 5 cortical columns each containing a population of excitatory and inhibitory cells, Bush and Sejnowski [35] found, in line with our results, that synchrony between the oscillations in both columns required connections from the excitatory cells of each column to the inhibitory cells of the other column. However, they did not systematically analyse all inter-columnar connection schemes or the effect of connection strength and, moreover, did not consider the situation where each column generated a different oscillation frequency. Tiesinga et al. (2001) [12] studied carbachol-induced transitions between oscillations of different frequencies in the hippocampus. Their simulations revealed delta oscillations interspersed with gamma oscillations, as well as theta oscillations interspersed with gamma oscillations, which emerged from a heterogeneous population of pyramidal (excitatory) cells: a strongly and a weakly interconnected subpopulation, where only the latter received projections from the (inhibitory) interneurons. Their study was not concerned with interactions between networks that on their own oscillated at a different frequency. Ainsworth et al. (2011) [33] built a computational model of the different gamma frequencies expressed in layer 2/3 and layer 4 of rat primary auditory cortex. They showed that the pattern of interlaminar connections may help stabilize frequency bifurcation caused by increased excitatory drive to this cortical region. Although several other authors have also studied systems of coupled oscillatory networks (e.g., [34], [36], [37], [38], each network is typically represented by a very few cells or else the mean field dynamics of the populations of excitatory and inhibitory neurons are described. As a consequence, firing synchrony between cells and hence oscillation power (amplitude) and frequency modulation cannot be investigated.

### Imposing source frequency onto target network

As mentioned, we found that the source network could impose its rhythm on the target network for high inter-network connection strength. In the hippocampus, interactions between oscillating networks may explain the change in frequency when connections from CA3 to CA1 are disrupted. In isolation, the CA1 expresses oscillations at a higher frequency than when the input from CA3 is intact [41], [54]. The CA3 and CA1 can independently generate oscillations [41], but the CA3 may impose its rhythm onto the CA1 via connections from CA3 to CA1. The frequency of hippocampal oscillations is determined by GABAA receptors, whose subunits are differentially expressed throughout the hippocampal circuitry. With connections from CA3 to CA1 intact, the α2-containing GABAA receptors in CA3 appear to determine the oscillation frequency in both CA3 and CA1 [43]. Without input from the CA3, the oscillation frequency in the CA1 may be driven by the faster α1-containing GABAA receptors expressed in the CA1.

Cortical oscillations have been postulated to play a role in the temporal binding of information [4], [16], [17]. According to this hypothesis, sensory neurons encoding different features of an object may synchronize their firing so as to indicate that their activity represent the same object. During motor maintenance behavior, for example, synchronized beta oscillations may bind spatially distributed sensory and motor representations [55]. During memory tasks, both oscillation power and coherence between hippocampal regions CA3 and CA1 are enhanced [43]. Our results suggest that slow, rather than fast, oscillations may be particularly well suited for synchronizing activity between cortical areas (see also [56]). Our study, as well as other modeling and experimental studies [41], [42], [46], also emphasizes the importance of strength and type of inter-network connectivity in achieving synchronization between neuronal networks. Indeed, impaired connectivity, as in neurological disorders such as schizophrenia, leads to abnormal neural oscillations and synchrony [57].

### Expression of two non-harmonic oscillation frequencies

Many intermediate situations between the temporal patterns presented here (Figs. 3h, 8–11) were found, but the examples shown clearly illustrate the range of complex temporal dynamics that can arise when one oscillating network connects to another oscillating network with a different oscillation frequency. In most temporal patterns in which two frequencies were co-expressed, frequencies alternated and/or fluctuated in power. Patterns such as those in Fig. 8, in which two frequencies were almost continually expressed, were relatively scarce.

Interestingly, the temporal dynamics of our model network shown in Figs. 9b and 11b, with the alternating expression of two distinct, non-harmonic oscillation frequencies, is qualitatively similar to the dynamics observed in layer 5 of rat PFC (Fig. 12). This suggests that the dynamics in the PFC may arise from the interaction between local subnetworks that each generate their own oscillation frequency. Indeed, layer 3/5 consistently oscillates at a higher frequency than layer 6, while layer 5 shows alternating episodes of slow and fast oscillations [26]. If we translated the connectivity scheme of Figs. 9b and 11b to the PFC situation, it would suggest that layer 6 pyramidal neurons project to layer 5 pyramidal neurons with moderate connection strength. Additionally, layer 5 pyramidal neurons would receive innervation from layer 6 interneurons.

What are the potential functional roles of the alternating expression of multiple oscillation frequencies? If the cortex makes use of different frequencies to process different aspects of incoming information, then activity may need to be kept separate in order to minimize interference. However, the cortex must at the same time possess ways of combining the information in these frequencies (see [23]). If in the two-network constellation, the slow oscillation encoded other information than the fast oscillation, then during episodes when both oscillations occur simultaneously in the target network, information contained in the slow and fast oscillations could be processed in parallel, whereas during episodes when either the slow or the fast oscillation is present, information could be processed separately (see also [26]). For example, in the PFC, layer 3/5, which generates fast oscillations, has been hypothesized to modulate the amount of alertness, whereas layer 6, which produces slow oscillations, may control the thalamic input to the PFC. Episodes during which both oscillation frequencies are simultaneously present in layer 5 may reflect parallel processing of both information streams [26].

### Induction of temporal fluctuations in oscillation power

As we have shown here, input from another oscillating network can not only change the frequency spectrum or cause the alternating expression of multiple frequencies in the target network, but also induce irregular fluctuations in oscillation power even when only a single frequency is expressed in the target network (Fig. 10). Fluctuations in oscillation power (amplitude) of ongoing oscillations, with irregular transitions between episodes of high- and low-amplitude oscillations, have been observed in many frequency bands and brain regions: in acute slices of rat prefrontal cortex [26], in alpha/beta oscillations in acute hippocampal slices [52], in theta/alpha oscillations in human EEG [28], and in alpha oscillations in human EEG [58]. Together with our previous findings [51] (see also [59]), this study suggests that amplitude fluctuations may arise from a temporary decrease in firing synchrony caused by the interference between network-generated oscillations and external input; the external input can come either in the form of random spike trains [51] or in the form of oscillating activity from another network (this study).

Episodes during which oscillation power (amplitude) is high reflect time periods of synchronized firing. Since synchronized firing between cells is important for Hebbian and spike-timing-dependent synaptic plasticity (STDP), high-amplitude episodes provide favorable conditions for altering synaptic strength. As we have shown here, afferent input from other oscillating networks can induce alternating episodes of high- and low-amplitude oscillations, thus modulating periods of learning and memory formation. In addition, afferent input can lead to the alternating expression of distinct frequencies, thereby determining during which frequencies synaptic strengths can change.

### Experimental testing of model results

Our model results on the impact of afferent oscillatory input on the frequency spectrum and temporal patterns of network oscillations, such as the alternating expression of multiple frequencies, could be tested experimentally in cortical or hippocampal slices cultured on multi-electrode arrays (MEAs). MEAs enable the recording of field potentials as well as the delivery of electrical signals with any desired temporal pattern. Thus, it can be tested how stimulating the network with oscillatory input, of a different frequency from that of the network's ongoing oscillations, changes the network's oscillation frequency and pattern. Alternatively, in the PFC, in which we hypothesized that the interaction between local subnetworks with different oscillation frequencies may lead to alternating expression of multiple frequencies, the strength of inter-network connections may be anatomically or pharmacologically modulated and the effects of these manipulations on oscillation pattern monitored. Yet another way to test our model predictions is to equalize the intrinsic oscillation frequencies in the PFC subnetworks by prolonging the IPSC decay in the slowest oscillating network by applying the GABAA-receptor modulator zolpidem [2], [60].

## Supporting Information

Figure S1 The iE connections from the slow to the fast network can impose the slow rhythm onto the fast network, albeit with moderate power. In all connectivity schemes, the fast network oscillated at the base frequency of the slow network for high iE connection strength. In addition, a frequency component close to the base frequency of the fast network could remain in the fast network even for high connections strengths (e.g., c4).
(TIF)

Figure S2 The iI connections from the slow to the fast network can impose the slow rhythm onto the fast network, albeit with moderate power. For moderate to high iI connection strengths, input from the slow network forced the fast network to oscillate at the base frequency (and/or its first harmonic) of the slow network. States with two different oscillation frequencies also occurred (d7, d8).
(TIF)

Figure S3 The Ei connections from the fast to the slow network strongly reduce oscillation power in the slow network. In all connectivity schemes, for high Ei connection strength, the input from the fast network strongly reduced the power of the oscillatory activity in the slow network, and was not able to entrain the slow network to the fast network. For low connection strengths, some entrainment to the fast network could occur (e.g., B3, B8) together with the presence of another frequency component (B8).
(TIF)

Figure S4 The Ie connections from the fast to the slow network strongly reduce oscillation power in the slow network. In all connectivity schemes, for high Ie connection strength, the input from the fast network strongly reduced the power of the oscillatory activity in the slow network, and was not able to entrain the slow network to the fast network. Patterns with two different frequencies appeared in some connectivity schemes (e.g., C6, C8) for low Ie connection strengths.
(TIF)

Figure S5 The Ii connections from the fast to the slow network can impose the fast rhythm onto the slow network, albeit with moderate power. For high Ii connection strength, the fast network could move the slow network to the base frequency of the fast network (D1, D4, D5, D7, D8). Patterns with two different frequencies appeared in some connectivity schemes (e.g., D7, D8) for low Ie connection strengths.
(TIF)

Figure S6 Effect of the number of connections from source to target network on the oscillatory activity in the target network. (P1–P4) Connectivity schemes from the slow to the fast network. (P5–P8) Connectivity schemes from fast to the slow network. Of the connection type depicted in red, the synaptic conductance was fixed at the indicated value of Cf, but the number of connected cells of that connection type was varied by changing its connection percentage. Entrainment of the target network to the source network occurred only for sufficiently high connection percentages (P1–P5), not at all (P6), or only for certain connection percentages (P7, P8). Once entrainment was established, the power of the frequency in the target network did (P1, P4, P5) or did not (P2, P3) strongly increase with connection percentage.
(TIF)

Text S1 Number of connections from source to target network also influences oscillation frequency in target network.
(DOC)
