# Supplementary figures and images for: Inter-Network Interactions: Impact of Connections between Oscillatory Neuronal Networks on Oscillation Frequency and Pattern
Source: PLoS One. 2014 Jul 9;9(7):e100899. doi: 10.1371/journal.pone.0100899 (PMC4090128; doi:10.1371/journal.pone.0100899)

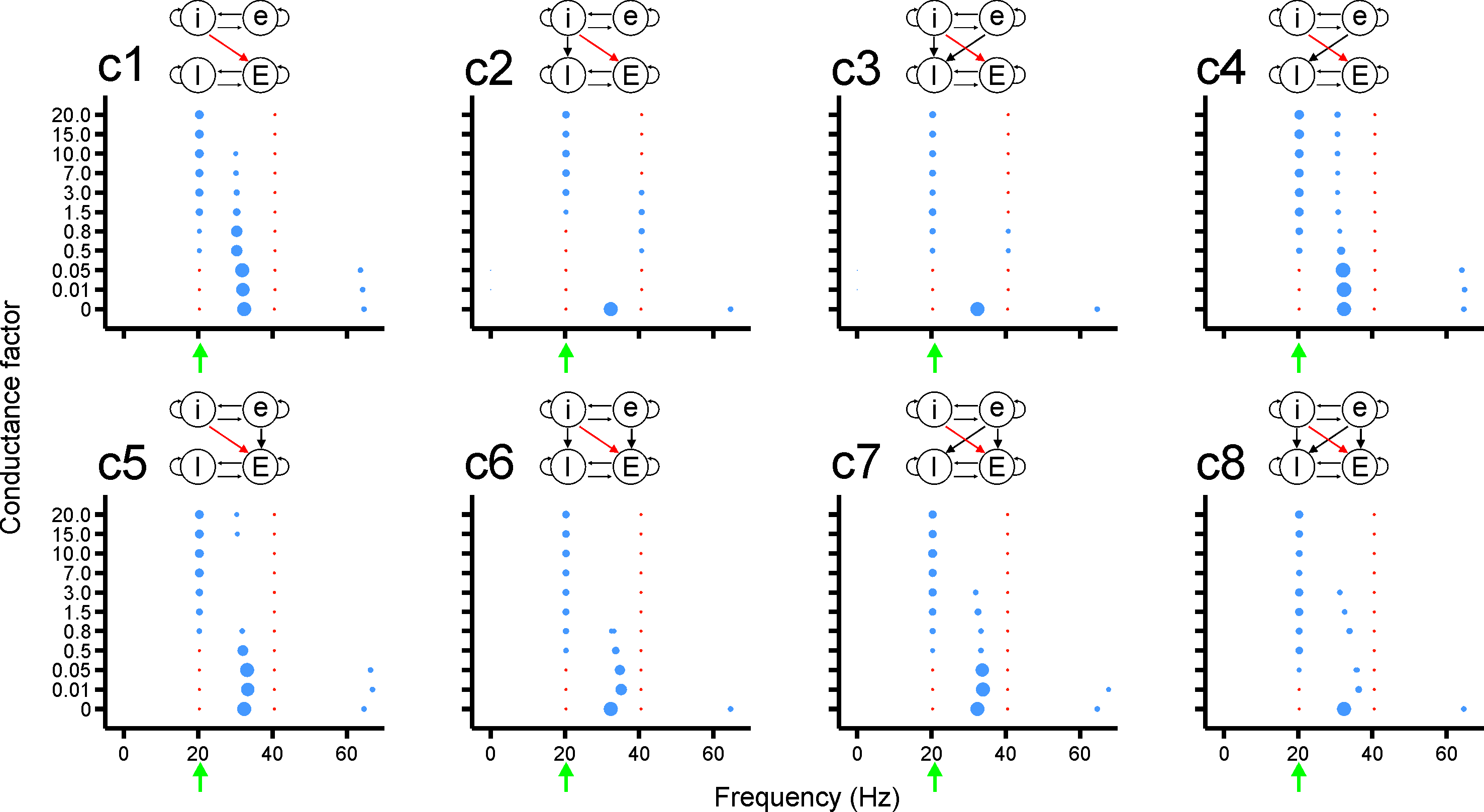

Supplement: Figure S1 — The iE connections from the slow to the fast network can impose the slow rhythm onto the fast network, albeit with moderate power. In all connectivity schemes, the fast network oscillated at the base frequency of the slow network for high iE connection strength. In addition, a frequency component close to the base frequency of the fast network could remain in the fast network even for high connections strengths (e.g., c4). (TIF) [file pone.0100899.s001.tif]

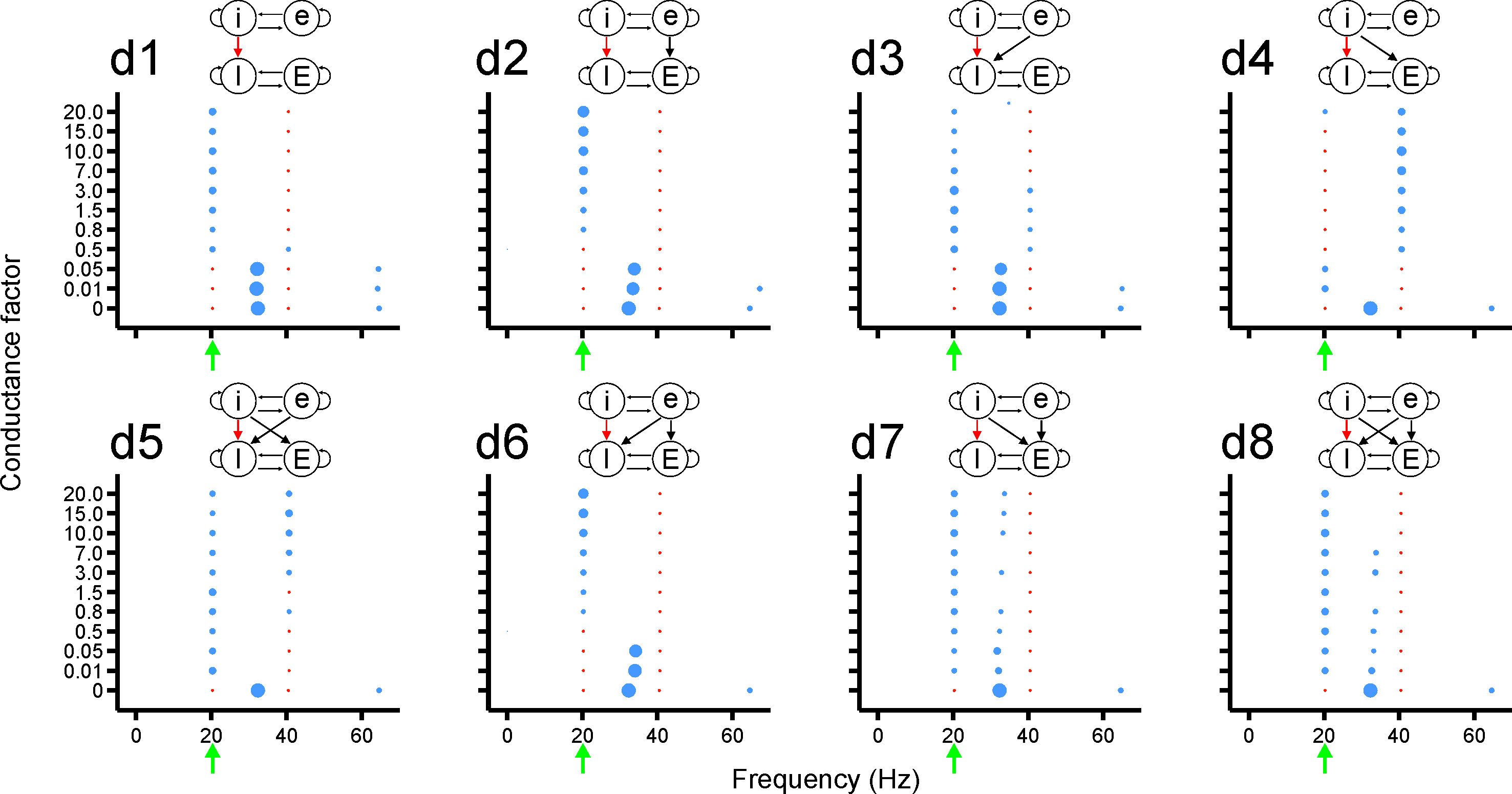

Supplement: Figure S2 — The iI connections from the slow to the fast network can impose the slow rhythm onto the fast network, albeit with moderate power. For moderate to high iI connection strengths, input from the slow network forced the fast network to oscillate at the base frequency (and/or its first harmonic) of the slow network. States with two different oscillation frequencies also occurred (d7, d8). (TIF) [file pone.0100899.s002.tif]

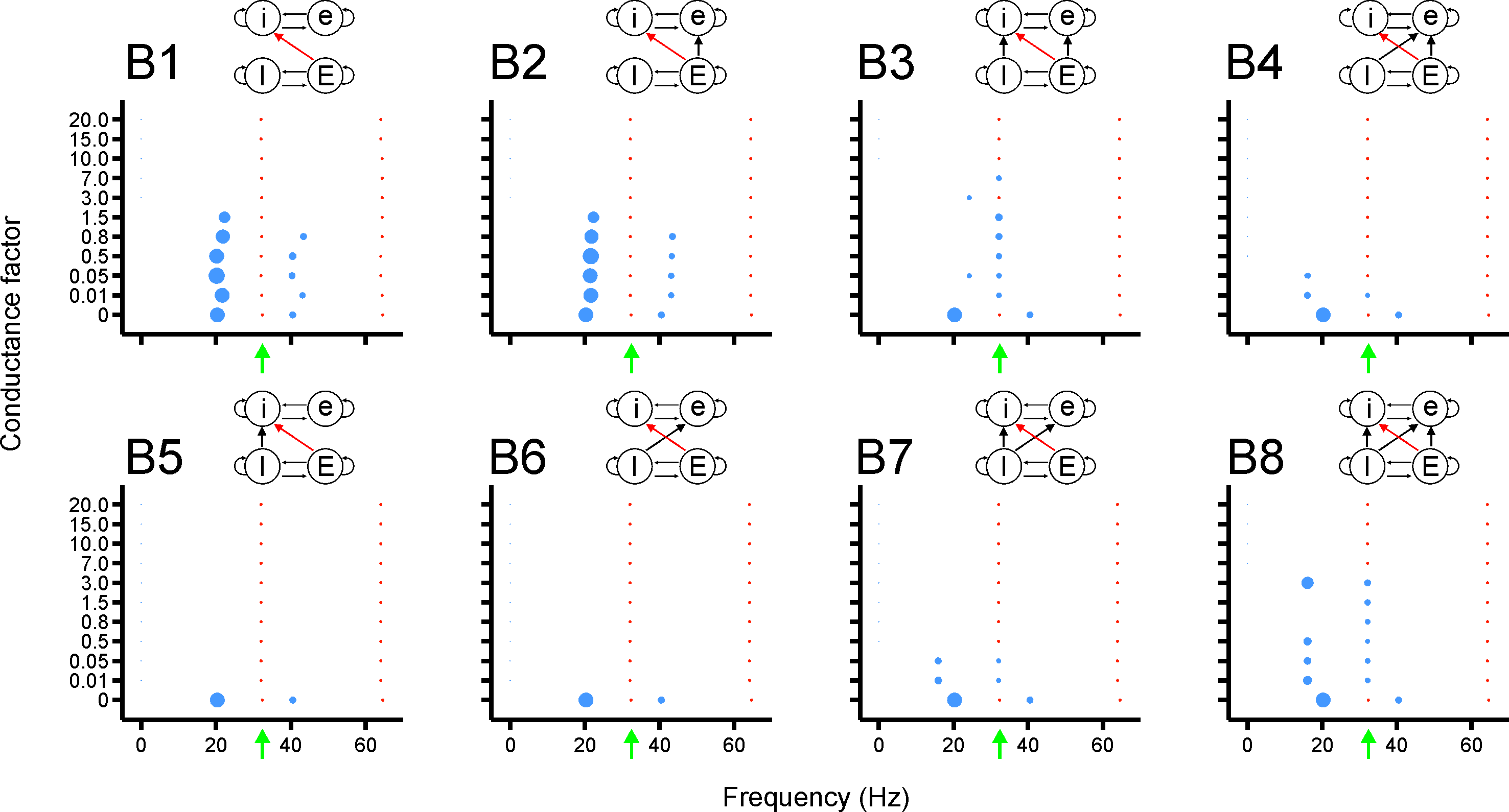

Supplement: Figure S3 — The Ei connections from the fast to the slow network strongly reduce oscillation power in the slow network. In all connectivity schemes, for high Ei connection strength, the input from the fast network strongly reduced the power of the oscillatory activity in the slow network, and was not able to entrain the slow network to the fast network. For low connection strengths, some entrainment to the fast network could occur (e.g., B3, B8) together with the presence of another frequency component (B8). (TIF) [file pone.0100899.s003.tif]

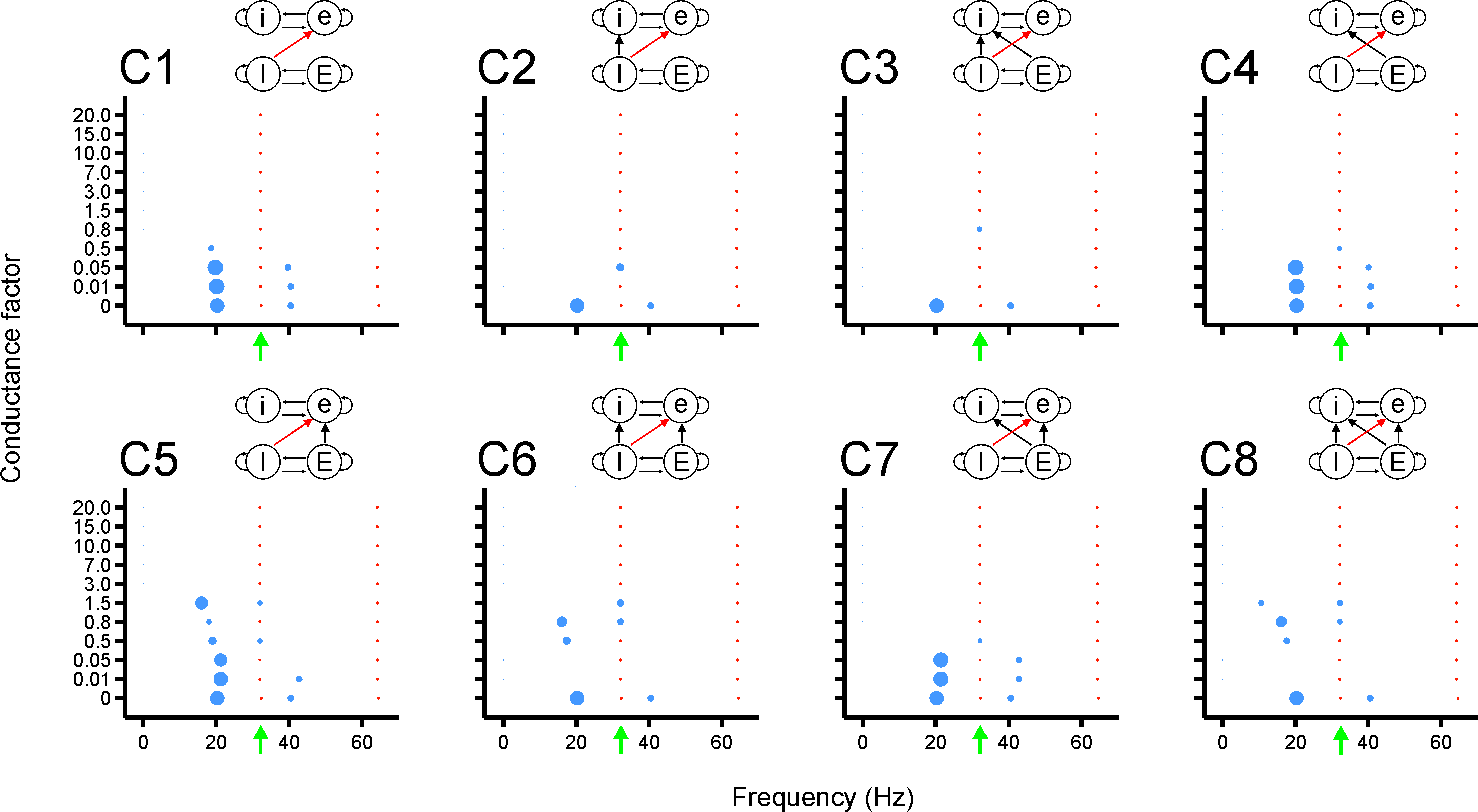

Supplement: Figure S4 — The Ie connections from the fast to the slow network strongly reduce oscillation power in the slow network. In all connectivity schemes, for high Ie connection strength, the input from the fast network strongly reduced the power of the oscillatory activity in the slow network, and was not able to entrain the slow network to the fast network. Patterns with two different frequencies appeared in some connectivity schemes (e.g., C6, C8) for low Ie connection strengths. (TIF) [file pone.0100899.s004.tif]

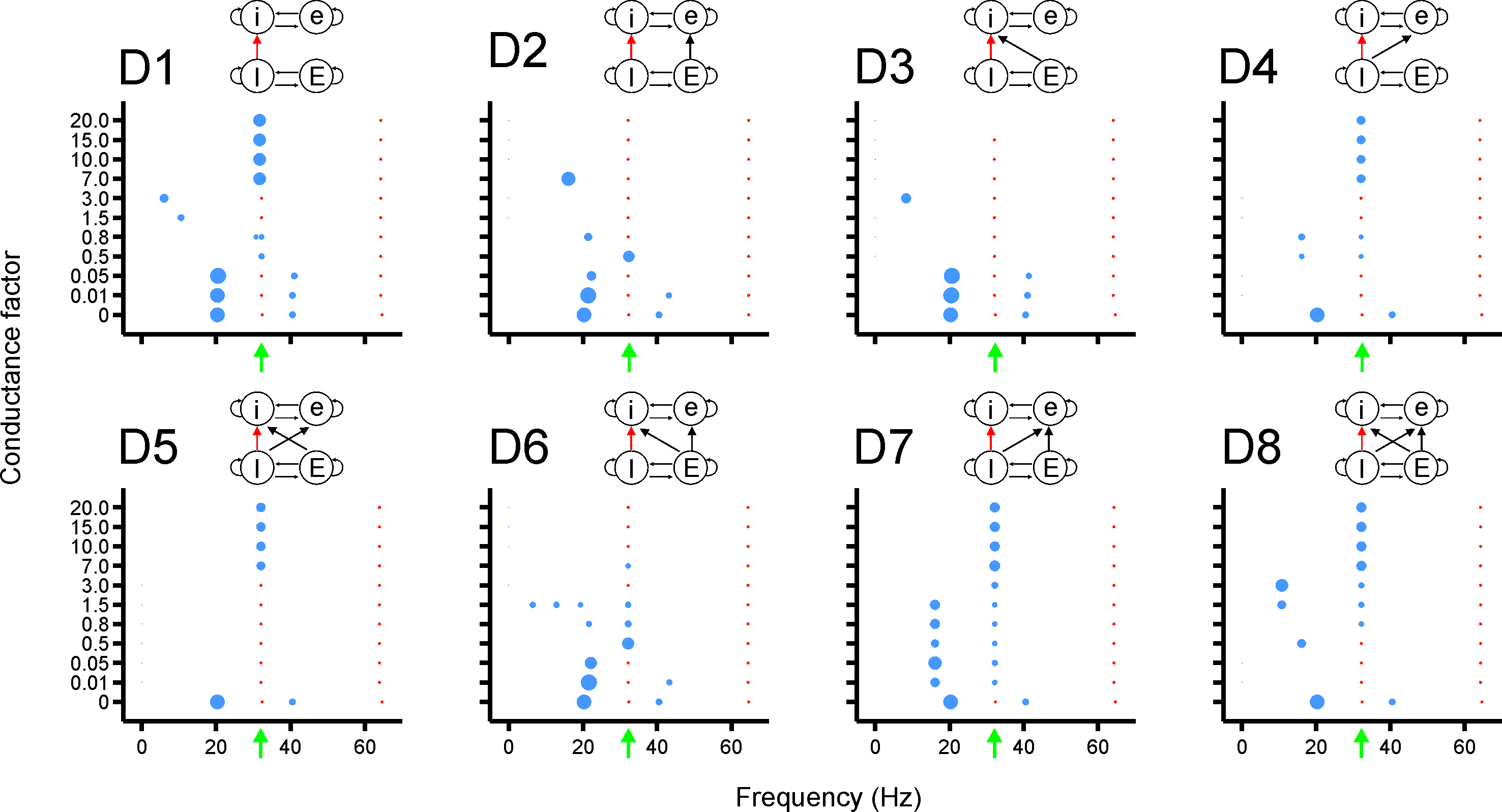

Supplement: Figure S5 — The Ii connections from the fast to the slow network can impose the fast rhythm onto the slow network, albeit with moderate power. For high Ii connection strength, the fast network could move the slow network to the base frequency of the fast network (D1, D4, D5, D7, D8). Patterns with two different frequencies appeared in some connectivity schemes (e.g., D7, D8) for low Ie connection strengths. (TIF) [file pone.0100899.s005.tif]

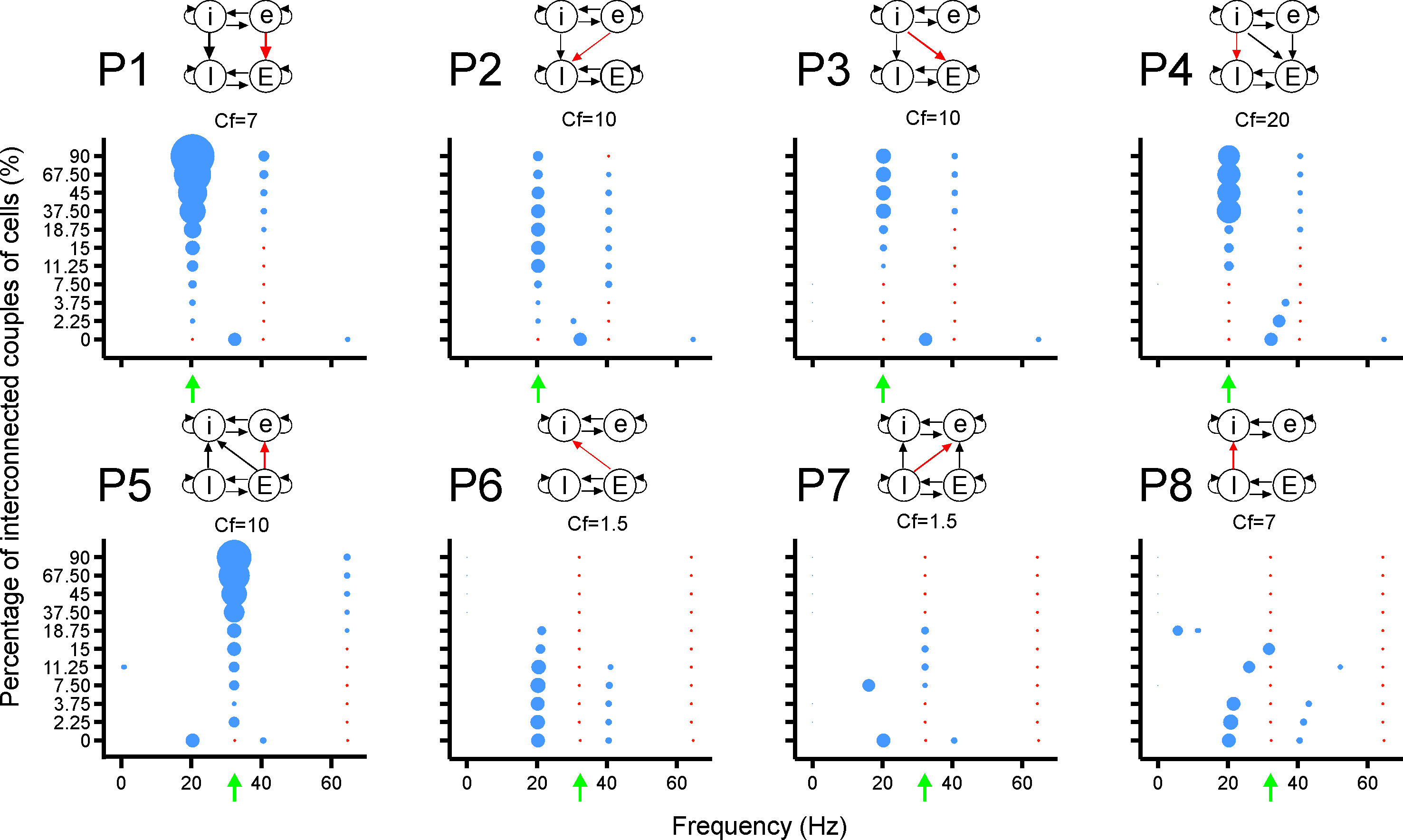

Supplement: Figure S6 — Effect of the number of connections from source to target network on the oscillatory activity in the target network. (P1–P4) Connectivity schemes from the slow to the fast network. (P5–P8) Connectivity schemes from fast to the slow network. Of the connection type depicted in red, the synaptic conductance was fixed at the indicated value of Cf, but the number of connected cells of that connection type was varied by changing its connection percentage. Entrainment of the target network to the source network occurred only for sufficiently high connection percentages (P1–P5), not at all (P6), or only for certain connection percentages (P7, P8). Once entrainment was established, the power of the frequency in the target network did (P1, P4, P5) or did not (P2, P3) strongly increase with connection percentage. (TIF) [file pone.0100899.s006.tif]
