# Supplementary material for: Inter-Network Interactions: Impact of Connections between Oscillatory Neuronal Networks on Oscillation Frequency and Pattern
Source: PLoS One. 2014 Jul 9;9(7):e100899. doi: 10.1371/journal.pone.0100899 (PMC4090128; doi:10.1371/journal.pone.0100899)
Supplement: Text S1 — Number of connections from source to target network also influences oscillation frequency in target network. (DOC) [file pone.0100899.s007.doc]

**Number of connections from source to target network also influences oscillation frequency in target network**

In the main text, we showed the effect of connection strength from the source to the target network on the oscillatory activity in the target network. The connection strength was varied by changing the excitatory or inhibitory synaptic conductance. When an excitatory or inhibitory population of the source network was connected to an excitatory or inhibitory population of the target network, not all the cells of the respective populations were connected, but only a fraction (see Methods). To illustrate that this fraction also had an impact on the ability of the source network to influence the dynamics of the target network, we varied it in four connectivity schemes from slow to fast network and in four connectivity schemes from fast to slow network (Fig. S6). In each connectivity scheme, we varied the fraction of the connection type under investigation (red arrows in Fig. S6), while keeping the fractions of the other connection types fixed. The probabilities for forming connections between the networks were by default 15% of the connection probabilities within each network (see Methods). For the connection type under investigation, we now varied this percentage between 0% and 90%.

With eE or eI connections from the slow to the fast network (Figs. S6P1, P2), the fast network became entrained to the slow network already at very low eE or eI connection percentages. With iE or iI connections (Figs. S6P3, P4), in contrast, much higher connection percentages were required. Once the rhythm in the fast network was locked to the frequency of the slow network, the power could strongly increase with increasing connection percentage (Fig. S6P1).

With Ee connections from the fast to the slow network (Fig. S6P5), the slow network became entrained to the fast network and the power increased with increasing connection percentage. In the other cases (Fig. S6P6-P8), the drive from the fast network suppressed the activity in the slow network for high connection percentages, so there was no entrainment (Fig. S6P6) or it disappeared (Fig. S6P7, P8) when the connection percentage was increased.

Thus, not only the connection strength (synaptic conductance) but also the number of connections from source to target network had an effect on the ability of the source network to influence the oscillatory dynamics in the target network. Changing the number of connections could thereby have a different effect on the dynamics than changing the connection strength (e.g., compare Fig. S6P8 and Fig. S5D1).
